# Microwave assisted synthesis of bis and tris(*ω***-**bromoacetophenones): versatile precursors for novel bis(imidazo[1,2**-***a*]pyridines), bis(imidazo[1,2**-***a*]pyrimidines) and their tris-analogs

**DOI:** 10.1186/1752-153X-7-105

**Published:** 2013-06-19

**Authors:** Mohamed R Shaaban

**Affiliations:** 1 Department of Chemistry, Faculty of Science, Cairo University, Giza, Egypt; 2 Department of Chemistry, Faculty of Applied Science, Umm Al-Qura University, Makkah Almukkarramah, Saudi Arabia

**Keywords:** Bromination, Bis(imidazo[1,2-*a*]pyridine), Bis(imidazo[1,2-*a*]pyrimidine), Microwaves

## Abstract

**Background:**

*α*-Bromination of the side chain of aromatic ketones using NBS in the presence of *p*-toluenesulfonic acid (*p*-TsOH) in acetonitrile is very common. However, regioselective bromination of bis and tris(*ω***-**bromoacetophenones) with NBS in the presence of *p*-TsOH in acetonitrile under microwave irradiation is quite novel. The bis- and tris(*ω***-**bromoacetophenones) are used in synthesis of bis and tris(heterocycles). bis(heterocycles) have received a great deal of attention, because many biologically active natural and synthetic products have molecular symmetry. The use of the pressurized microwave irradiation is very advantageous to many syntheses and provide a large rate enhancement.

**Results:**

Bis and tris(*ω***-**bromoacetophenones) were obtained as single monobrominated derivatives in a shorter time than the conventional conditions. The results clearly demonstrate the better reactivity and selectivity of NBS/*p*-TsOH/CH_3_CN as a brominating mixture under microwave conditions. The reaction of bis and tris(*ω*-bromoacetophenone) with 2-aminopyridine and 2-aminopyrimidine proceeded smoothly in a mixture of anhydrous ethanol and DMF under reflux or using 300 W/105°C/ 20 min microwave irradiation conditions to afford the corresponding bis(imidazo[1,2-*a*]pyridine), bis(imidazo[1,2-*a*]pyrimidine) and tris(imidazo[1,2-*a*]pyridine) derivatives in moderate to excellent yields. The carbonyl analogue of the targeted bis(imidazopyridines) could be synthesized by the reaction of *N*,*N*-dimethyl-*N'*-(pyridin-2-yl)formimidamide with bis(*ω*-bromoacetophenone) in refluxing ethanol. The structures of the newly synthesized compounds were confirmed by their spectral data as well as their elemental analyses.

**Conclusion:**

In conclusion, selective *α*-bromination of bis- and tris(acetophenones) has been accomplished efficiently utilizing NBS/*p*-TsOH/CH_3_CN under microwave irradiation. In addition, a facile synthesis of novel series of bis- and tris(imidazopyridine) and bis(imidazopyrimidine) derivatives.

## Background

In recent decades, imidazo[1,2-*a*]pyridine and the related imidazo[1,2-*a*]pyrimidines derivatives have received a significant attention in pharmaceutical industry owing to their interesting biological activities [1]. They displayed a broad range of therapeutic activities, including antibacterial [2], antifungal [3], antiviral [4-6], and inhibitors of p38MAP kinase [7]. They have also been used as inotropic and *β*-blocking agents [8], benzodiazepine receptor agonists [9], and anesthetic activity [10]. Drug formulations containing imidazo[1,2-*a*]pyridines are currently available on the market, for example alpidem (anxiolytic), zolpidem (hypnotic), and zolimidine (antiulcer). On the other hand, bis(heterocycles) have received a great deal of attention, not only as model compounds for main chain polymers but also because many biologically active natural and synthetic products have molecular symmetry [11-22]. Moreover, the synthesis of the imidazo[1,2-*a*]pyridine and imidazo[1,2-*a*]pyrimidine ring systems has been widely investigated [23-31]. One of the most common strategies uses 2-aminopyridine or 2-aminopyrimidine, and *α*-halocarbonyl compound as starting materials. However, up to the best of our knowledge no synthesis of bis(imidazo[1,2-*a*]pyridine) and bis(imidazo[1,2-*a*]pyrimidine) ring systems were found in the literature, so far even under conventional conditions. Nowadays, the use of the pressurized microwave irradiation can be very advantageous to many chemistries where the solvent can be heated up to temperatures that are 2–4 times their respective boiling points and thus providing large rate enhancement [32-34]. In addition, keeping the atmosphere away from moisture that may affect the moisture sensitive reagents decreases the possibility of formation of the undesired byproducts. As a part of systematic interest in the synthesis of fused heterocyclic systems having potential unique properties [35-38], and in continuation to our interest in the synthetic utility of bis(*ω*-bromoacetophenones) as building blocks for novel bis(fused-heterocycles) [39], the aim of the present work is to define versatile route to synthesize bis- and tris(fused-heterocycles), in an efficient one step synthesis under microwave irradiation. Also, *α*-bromination of a structurally different acetophenones using NBS and toluenesulfonic acid as an inexpensive catalyst in acetonitrile under microwave irradiation was improved.

## Results and discussion

**Scheme 1:**
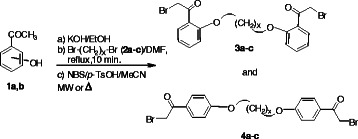
Synthesis of bis(*ω*-bromoacetophenones) 3a-c and 4a-c **Synthesis of bis(*ω*-bromoacetophenones) 3a-c and 4a-c.**

*α*-Bromination of the side chain of aromatic ketones has attracted attention because the resulting bromoketones are important synthons used for the variety of biologically active heterocyclic compounds. Recently, we reported the synthesis of bis(*ω***-**bromoacetophenones) using the reaction of their corresponding bis(acetophenones) with NBS in the presence of *p*-toluenesulfonic acid (*p*-TsOH) in acetonitrile under conventional heating. However the long reaction time was one of the disadvantages at that time [39]. *ω***-**Bromoacetophenones **3a-c** and **4a-c** were obtained as single monobrominated derivatives in a shorter time than the conventional conditions (Scheme 1, Table 1). The results clearly demonstrate the better reactivity and selectivity of NBS/*p*-TsOH/CH_3_CN as a brominating mixture under microwave conditions, in fact dibromination or aromatic ring bromination were not observed.

**Table 1:** Yield % of the synthesized bis(*ω*-bromoacetophenones) 3a-c and 4a-c

**Entry**	**X**	**3a-c( *o- *isomer)**	**Yield%**^**a,[39]**^	**Yield%**^**a**^
			**∆**	**MW**
1	2	**3a**	68	74
2	3	**3b**	83	86
3	4	**3c**	92	95
		**4a-c( *p- *isomer)**		
4	2	**4a**	57	60
5	3	**4b**	85	89
6	4	**4c**	95	95

^a^Yields of isolated compounds.

**Scheme 2:**
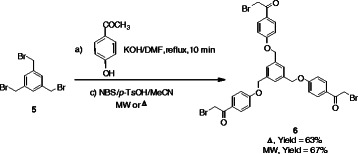
**Synthesis of tris(*ω*-bromoacetophenones) 6.**

In the same manner, the structurally interesting tripodal *ω*-bromoacetophenone derivative **6** could be obtained by the reaction of the potassium salt of 4-hydroxyacetophenone (**1b**) with the appropriate 1,3,5-tris(bromomethyl)benzene **5**, in boiling DMF followed by NBS bromination under microwave irradiation (Scheme 2). The structure of the tris(*ω*-bromoacetophenone) derivative **6** was confirmed by its elemental analyses and spectral data. For example, the ^1^H NMR spectra of **6** displayed a singlet signal at δ 4.40 due to CH_2_ protons, a singlet signal at 5.19 due to CH_2_O protons, two doublets at δ 7.01 and 7.93 (*J* = 9.0 Hz) due to aromatic protons, in addition to core aromatic ring protons singlet at δ 7.49.

**Scheme 3:**
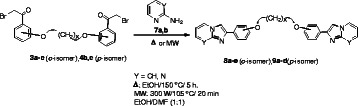
Synthesis of bis(imidazo[1,2-*a*]pyridine) and bis(imidazo[1,2-*a*]pyrimidine) derivatives 8a-e and 9a-d **Synthesis of bis(imidazo[1,2-*a*]pyridine) and bis(imidazo[1,2-*a*]pyrimidine) derivatives 8a-e and 9a-d.**

Several routes for imidazo[1,2-*a*]pyridine and imidazo[1,2-*a*]pyrmidine skeletons have been developed [23-31], but, the access by the reported methods to those functionalized fused systems, is usually difficult. In this context, 2-aminoazines were chosen as bis(nucleophilic) heterocyclic reagents. These heterocyclic amines namely, 2-aminopyridine (**7a**) and 2-aminopyrimidine (**7b**) have readily accessible two nucleophilic centers for the preparation of bis(imidazo[1,2-*a*]pyridine) and bis(imidazo[1,2-*a*]pyrimidine) derivatives, respectively. The reaction of bis(*ω*-bromoacetophenone) **3a-c** and **4b,c** with 2-aminopyridine and 2-aminopyrimidine (**7a,b**) proceeded smoothly in a mixture of anhydrous ethanol and DMF under reflux or using 300 W/105°C/ 20 min microwave irradiation conditions to afford the corresponding bis(imidazo[1,2-*a*]pyridine) and bis(imidazo[1,2-*a*]pyrimidine) derivatives **8a-e** and **9a-d** in good to excellent yields (Scheme 3, Table 2).

**Table 2:** **Yield % of the synthesized bis(imidazo[1,2-*a*]pyridine) and bis(imidazo[1,2-*a*]pyrimidine) derivatives 8a-e and 9a-d**

**Entry**	**X**	**Y**	**8a-e( *o- *isomer)**	**Yield%**^**a**^	**Yield%**^**a**^
				**Δ**	**MW**
1	2	CH	**8a**	78	81
2	3	CH	**8b**	69	74
3	4	CH	**8c**	72	89
4	2	N	**8d**	53	70
5	3	N	**8e**	63	83
			**9a-d( *p- *isomer)**		
6	3	CH	**9a**	55	82
7	4	CH	**9b**	65	87
8	3	N	**9c**	68	78
9	4	N	**9d**	64	86

^a^Yields of isolated compounds.

The structure of the products **8a-e** and **9a-d** were confirmed by their spectral data as well as their elemental analyses. For example, the disappearance of bands attributed C = O stretching frequency in the ir spectra is a good evidence for the structure given to those compounds. The ^1^H NMR spectra of compounds **8a-e** and **9a-d** showed a characteristic singlet signal resonance around *δ* 8.50 due to (=C**-**H) of the imidazole ring (Additional file 1). In addition, the pyridine or pyrimidine ring protons were seen at the expected chemical shifts and integral values.

**Scheme 4:**
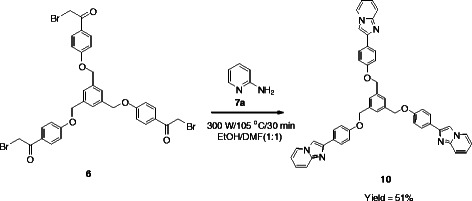
**Synthesis of tris(imidazo[1,2-*a*]pyridine) 10.**

In the same manner when the tris(*ω*-bromoacetophenone) **5** was treated with 2-aminopyridine (**7a**), under the same microwave conditions, it afforded the tripodal imidazo[1,2-*a*]pyridine **10** (Scheme 4), however the yield of the product was moderate after 30 min. of irradiation.

**Scheme 5:**
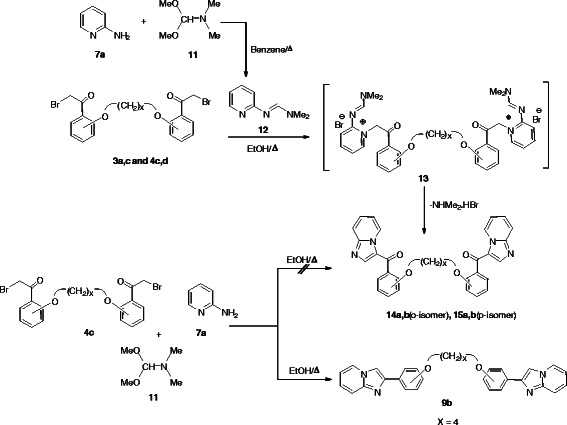
Synthesis of the carbonyl analogue of the targeted bis(imidazoazines) Synthesis of the carbonyl analogue of the targeted bis(imidazoazines).

The carbonyl analogue of the targeted bis(imidazopyridines) **14a,b** and **15a,b** could be synthesized by the reaction of *N*,*N*-dimethyl-*N'*-(pyridin-2-yl)formimidamide **12** with bis(*ω*-bromoacetophenone) **3a,c** and **4c,d** in refluxing ethanol (Scheme 5, Table 3).

**Table 3:** Yield % of the synthesized carbonyl analogue of the targeted bis(imidazoazines)

**Entry**	**X**	**14,b( *o- *isomer)**	**Yield%**^**a**^
1	2	**14a**	58
2	4	**14b**	60
		**15-d( *p *-isomer)**	
3	3	**15a**	78
4	4	**15b**	81

^a^Yields of isolated compounds.

**Figure 1:**
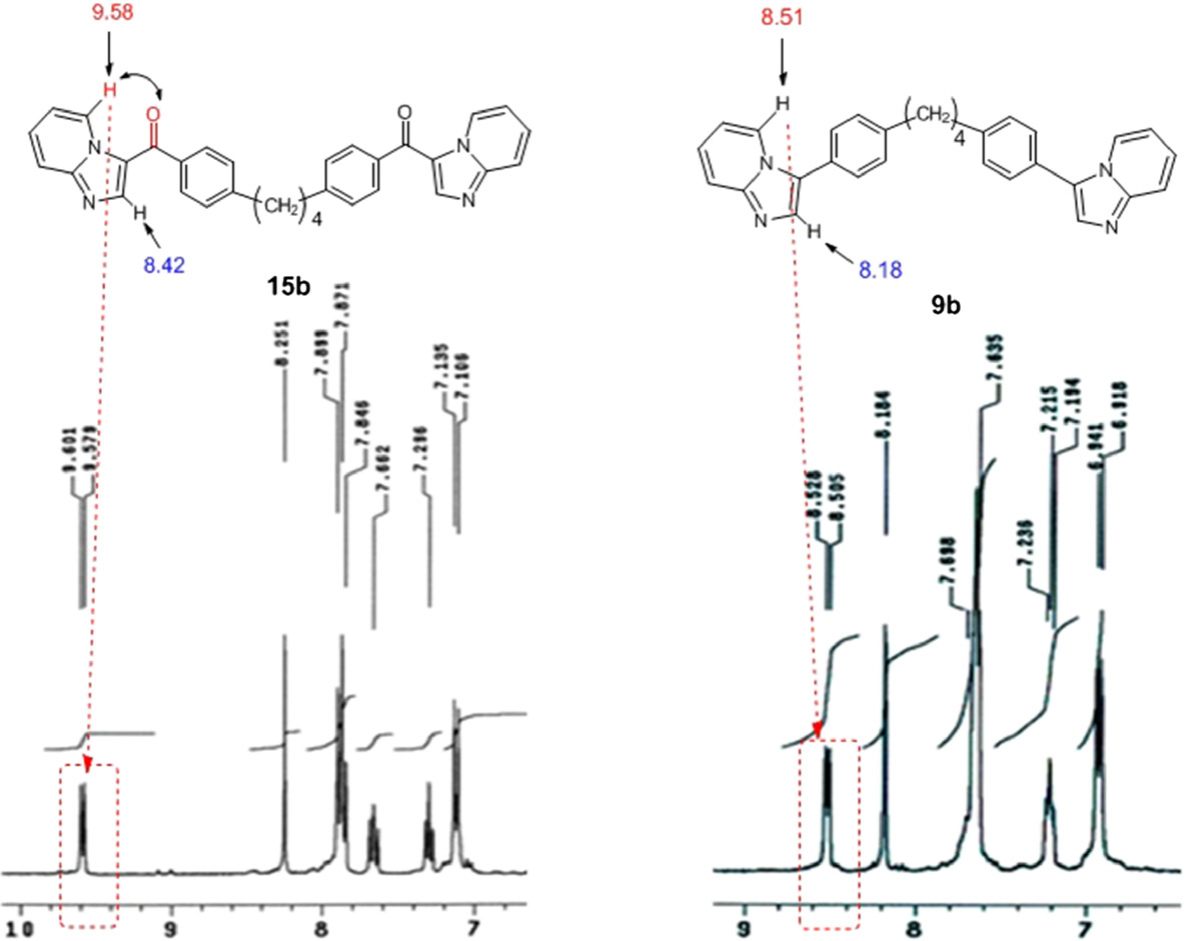
**Aromatic region of the **^**1**^**H NMR spectra of the *p*-isomers of 15b and 9b.**

The IR spectrum of compound **15a** (taken as an example) in KBr showed, in addition to the expected peaks of the imidazopyridines **15a**, a peak at wave numbers near 1660 cm^-1^ corresponding to the C = O group. The presence C = O band was an evidence for the pathway of the cyclocondensation of the appropriate bis(*ω*-bromoacetophenone) derivatives with the *N*,*N*-dimethyl-*N'*-(pyridin-2-yl)formimidamide **12** as shown in Scheme 5. Moreover, the ^1^H NMR spectra of the *p*-isomers of imidazoazines **15** showed that C5-*H* signal of **15b** is downfield of the corresponding proton for **9b**, respectively which provide an additional support for the suggestion that the carbonyl group anisotropy impacts the position of the aforementioned NMR signals as shown in Figure 1.

It was expected that the bis(imidazopyridine) **15b** could be obtained *via* the alternative one pot three component reaction of the bis(*ω*-bromoacetophenone) derivative **4c** (taken as an example), 2-aminopyridine (**7a**), and dimethylformamide dimethylacetal (**11**) under solvent free conditions. However, the reaction afforded a product identical in all respects (mp, mixed mp, and spectra) with the analogue imidazopyridine **9b** rather than **15b** as shown in Scheme 5.

## Conclusion

In conclusion, selective *α*-bromination of bis- and tris(acetophenones) has been accomplished efficiently utilizing NBS/*p*-TsOH/CH_3_CN under microwave irradiation. In addition, a facile synthesis of novel series of bis- and tris(imidazopyridine) and bis(imidazopyrimidine) derivatives *via* the reaction of bis(*ω*-bromoacetophenone) derivatives with the appropriate aminoazine or their formamidine derivatives was achieved. The synthesized bis-, and tris(fused-heterocycles) offer an advantage of their easy synthesis on a large scale quantities in a simple procedure from inexpensive starting materials and it is expected that they would be useful compounds with potentially high pharmacological and biological activities.

## Experimental section

### Materials and equipments

All melting points were measured on a Gallenkamp melting point apparatus. The infrared spectra were recorded in potassium bromide discs on a Pye Unicam SP 3–300 and Shimadzu FT IR 8101 PC infrared spectrophotometers. The NMR spectra were recorded on a Varian Mercury VXR-300 NMR spectrometer (^1^H NMR (300 MHz) and ^13^C NMR (75.46 MHz)) and Bruker-500 NMR spectrometer (^1^H NMR (500 MHz) and ^13^C NMR (125.77 MHz)) were run in deuterated chloroform (CDCl_3_) or dimethyl sulfoxide (DMSO-*d*_*6*_). Chemical shifts were related to that of the solvent. Mass spectra were recorded on a Shimadzu GCMS-QP1000 EX mass spectrometer at 70 eV. Elemental analyses were carried out at the Micro-analytical Centre of Cairo University, Giza, Egypt and recorded on Elementar-Vario EL automatic analyzer. Microwave irradiation was performed using the MARS system of CEM which is a multi-mode platform equipped with a magnetic stirring plate and a rotor that allows the parallel processing of several vessels per batch. We used the HP-500 (teflon (TFA) insert) (vessel volume 80 mL, max pressure 350 psi, max temperature 210°C) in order to get the maximum save operation. 1,*ω*-bis(4-acetylphenoxy)alkane and tris(acetophenone) derivatives were prepared following the reported procedures [39-41].

### Synthesis and characterization

#### bis(*ω*-bromoacetophenone) derivatives 3a-c, 4a-c and 6

##### General procedure

### Thermal method

The appropriate bis(acetylphenoxy)alkanes or Tris(acetylphenoxy)alkane (l0 mmol) and *p*-toluenesulphonic acid (*p*-TsOH) (5.6 g, 20 mmol), in MeCN (50 mL), was slowly added *N*-bromosuccinimide (NBS) (3.6 g, 20 mmol). After addition of NBS was complete, the reaction mixture was refluxed with stirring for 2–3 h then left to cool to room temperature. The solvent was evaporated *in vacuo* and the residue was dissolved in CHCl_3_ (50 mL), washed with water (3 × 20 mL) and dried over MgSO_4_. After evaporation of the solvent, the resulting solid was recrystallized from benzene to afford the corresponding bis and tris(*ω*-bromoacetophenone) derivatives **3a-c 4a-c** and **6**, respectively [39].

### Microwave method

The appropriate bis(acetylphenoxy)alkanes or Tris(acetylphenoxy)alkane (10 mmol) and *p*-TsOH (20 mmol) were mixed in a HP-500 process vial and NBS (10 mmol) was added portionwise with stirring for 5 min. before irradiation. The vial was capped properly and irradiated by microwaves using pressurized conditions at 90°C for 20–30 min. The solvent was evaporated *in vacuo* and the residue was dissolved in CHCl_3_ (50 mL), washed with water (3 × 20 mL) and dried over MgSO_4_. After evaporation of the solvent, the resulting solid was recrystallized from benzene to afford the corresponding bis and tris(*ω*-bromoacetophenone) derivatives **3a-c**, **4a-c** and **6**, respectively. The physical and spectral data of the newly synthesized tris(*ω*-bromoacetophenone) 6 are listed below.

**6**: Yield 63% (thermally) and 67% (under microwave irradiation), mp. 130°C; IR (KBr) ν_max_/cm^-1^ 1672 (C = O); ^1^H NMR (DMSO-*d*_*6*_) δ 4.40 (s, 6H), 5.19 (s, 6H), 7.02 (d, 6H, *J* = 9 Hz), 7.49 (s, 3H), 7.96 (d, 6H, *J* = 9 Hz). MS *m/z* 765 (M^+^+6), 763 (M^+^+4), 761 (M^+^+2), 759 (M^+^). For C_33_H_27_Br_3_O_6_ Calcd: C, 52.20; H, 3.58. Found: C, 52.22; H, 3.56.

#### Bis(imidazopyridine) and bis(imidazopyrimidine) derivatives 8a-e, 9a-d and 10

##### General procedure

### Thermal method

A mixture of the appropriate bis(*ω*-bromoacetophenone) derivatives **3a-c** or **4a,b** (5 mmol), 2**-**aminopyridine or 2-aminopyrimidine **7a,b** (10 mmol) in absolute EtOH, was heated at refluxing temperature for 5h. The reaction mixture was then left to cool and the resulting solid was collected by filtration, washed thoroughly with ethanol and dried. Recrystallization from DMF afforded the corresponding bis(fused-heterocyclic) derivatives **8a-e** and **9a-d**, respectively.

### Microwave method

A mixture of the appropriate bis(*ω*-bromoacetophenone) or tris(*ω*-bromoacetophenone) derivatives **3a-c**, **4a,b** or **6** (5 mmol), 2-aminopyridine or 2-aminopyrimidine **7a,b** (10 mmol) in EtOH/DMF(1:1) were mixed in a HP-500 process vial. The vial was capped properly and irradiated by microwaves using pressurized conditions at 105°C for 20–30 min. The reaction mixture was then left to cool and the resulting solid was recrystallized from DMF to afford the corresponding bis(fused-heterocyclic) derivatives **8a-e**, **9a-d** and **10**, respectively. The physical and spectral data of the newly synthesized compounds are listed below.

**8a**: mp 293–295°C; IR (KBr) ν_max_/cm^-1^ 1605 (C = N); ^1^H NMR (DMSO-*d*_*6*_) δ 4.82 (s, 4H), 7.14-7.17 (t, 2H, *J* = 9 Hz), 7.35-7.37 (t, 2H, *J* = 9 Hz), 7.43-7.45 (d, 2H, *J* = 9 Hz), 7.50-7.52 (t, 2H, *J* = 9 Hz), 7.73-7.75 (d, 2H, *J* = 9 Hz), 7.81-7.87 (m, 4H, *J* = 9 Hz), 8.54 (s, 2H), 8.55-8.57 (d, 2H, *J* = 9 Hz); ^13^C NMR (DMSO-*d*_*6*_) δ 67.64, 111.69, 112.13, 113.54, 114.06, 115.29, 116.83, 121.40,127.66, 128.21, 131.56, 132.95, 139.03, 155.84. MS *m/z* 446 (M^+^). For C_28_H_22_N_4_O_2_ Calcd: C, 75.32; H, 4.97; N, 12.55. Found: C, 75.29; H, 4.95; N, 12.49.

**8b**: mp. 258–260°C; IR (KBr) ν_max_/cm^-1^ 1605 (C = N); ^1^H NMR (DMSO-*d*_*6*_) δ 2.51-2.61 (br, 2H), 4.48-4.49 (br, 4H), 7.03-7.06 (t, 2H, *J* = 9 Hz), 7.26-7.27 (d, 2H, *J* = 9 Hz), 7.39-7.42 (t, 2H, *J* = 9 Hz), 7.48-7.51 (m, 2H, *J* = 9 Hz), 7.85-7.87 (d, 2H, *J* = 9 Hz), 7.88-7.94 (m, 4H, *J* = 9 Hz), 8.70 (s, 2H), 9.02-9.03 (d, 2H, *J* = 9 Hz); ^13^C NMR (DMSO-*d*_*6*_) δ 27.84, 66.00, 111.85, 113.00, 113.49, 114.85, 116.78, 120.79,127.71, 128.96, 131.44, 131.51, 133.10, 139.24, 155.84. MS *m/z* 460 (M^+^). For C_29_H_24_N_4_O_2_ Calcd: C, 75.63; H, 5.25; N, 12.17. Found: C, 75.66; H, 5.23; N, 12.18.

**8c**: mp. 267–268°C; IR (KBr) ν_max_/cm^-1^ 1605 (C = N); ^1^H NMR (DMSO-*d*_*6*_) δ 2.16-2.18 (br, 4H), 4.37-4.39 (br, 4H), 7.08-7.12 (t, 2H, *J* = 9 Hz), 7.20-7.22 (d, 2H, *J* = 9 Hz), 7.36-7.38 (t, 2H, *J* = 9 Hz), 7.47-7.48 (m, 2H, *J* = 9 Hz), 7.88-7.90 (d, 2H, *J* = 9 Hz), 7.90-7.92 (m, 4H, *J* = 9 Hz), 8.63 (s, 2H), 8.95-8.97 (d, 2H, *J* = 9 Hz); ^13^C NMR (DMSO-*d*_*6*_) δ 25.38, 68.27, 111.89, 112.80, 113.43, 114.80, 116.81, 120.72, 127.73, 128.90, 131.38, 131.71, 133.09, 139.29, 155.78. MS *m/z* 474 (M^+^). For C_30_H_26_N_4_O_2_ Calcd: C, 75.93; H, 5.52; N, 11.81. Found: C, 75.91; H, 5.50; N, 11.83.

**8d**: mp. 293–294°C; IR (KBr) ν_max_/cm^-1^ 1600 (C = N); ^1^H NMR (DMSO-*d*_*6*_) δ 4.81 (s, 4H), 7.15-7.17(t, 2H, *J* = 9 Hz), 7.31-7.33(t, 2H, *J* = 9 Hz), 7.43-7.45 (d, 2H, *J* = 9 Hz), 7.50-7.52(t, 2H, *J* = 9 Hz), 7.71-7.73 (d, 2H, *J* = 9 Hz), 7.86-7.88 (m, 2H), 8.50-8.50 (m, 4H); ^13^C NMR (DMSO-*d*_*6*_) δ 67.61, 111.83, 113.53, 114.02, 116.66, 121.39, 127.67, 128.13, 131.47, 132.68, 139.16, 145.36, 155.83. MS *m/z* 448 (M^+^). For C_26_H_20_N_6_O_2_ Calcd: C, 69.63; H, 4.49; N, 18.74. Found: C, 69.61; H, 4.51; N, 19.72.

**8e**: mp. 257–259°C; IR (KBr) ν_max_/cm^-1^ 1608 (C = N); ); ^1^H NMR (DMSO-*d*_*6*_) δ 2.61-2.64 (m, 2H), 4.48-4.50 (m, 4H), 7.03-7.06(t, 2H, *J* = 9 Hz), 7.26-7.27(d, 2H, *J* = 9 Hz), 7.38-7.42 (t, 2H, *J* = 9 Hz), 7.46-7.49 (t, 2H, *J* = 9 Hz), 7.89-7.95 (m, 4H), 8.71(s, 2H), 9.03-9.04 (d, 2H, *J* = 9 Hz); ^13^C NMR (DMSO-*d*_*6*_) δ 27.83, 65.90, 111.96, 112.89, 113.39, 115.10, 116.49, 120.71, 127.68, 128.82, 131.24, 132.67, 139.37, 155.59. MS *m/z* 462 (M^+^). For C_27_H_22_N_6_O_2_ Calcd: C, 70.12; H, 4.79; N, 18.17. Found: C, 70.14; H, 4.76; N, 18.14.

**9a**: mp. 280–281°C; IR (KBr) ν_max_/cm^-1^ 1605 (C = N); ^1^H NMR (DMSO-*d*_*6*_) δ 2.26-2.28 (br, 2H), 4.25-4.29 (br, 4H), 7.19-7.21 (d, 4H, *J* = 9 Hz), 7.45-7.48 (t, 2H, *J* = 9 Hz), 7.87-7.95 (m, 6H, *J* = 9 Hz), 8.76 (s, 2H), 8.90-8.89 (d, 4H, *J* = 9 Hz); ^13^C NMR (DMSO-*d*_*6*_) δ 28.42, 64.88, 109.83, 113.36, 115.27, 116.74, 119.23, 127.67, 128.64, 132.36, 136.02, 140.16, 160.17. MS *m/z* 460 (M^+^). For C_29_H_24_N_4_O_2_ Calcd: C, 75.63; H, 5.25; N, 12.17. Found: C, 75.61; H, 5.23; N, 12.15.

**9b**: mp. 235–236°C; IR (KBr) ν_max_/cm^-1^ 1608 (C = N); ^1^H NMR (DMSO-*d*_*6*_) δ 1.91-1.92 (br, 4H), 4.11-4.18 (br, 4H), 7.08-7.10 (d, 4H, *J* = 9 Hz), 7.59-7.60 (t, 2H, *J* = 9 Hz), 7.84-7.99 (m, 6H, *J* = 9 Hz), 8.35 (s, 2H), 8.55-8.56 (d, 4H, *J* = 9 Hz); ^13^C NMR (DMSO-*d*_*6*_) δ 25.23, 66.59, 109.76, 114.03, 114.85, 117.15, 122.77, 126.81, 128.15, 130.94, 131.04, 148.07, 161.69. MS *m/z* 474 (M^+^). For C_30_H_26_N_4_O_2_ Calcd: C, 75.93; H, 5.52; N, 11.81. Found: C, 75.90; H, 5.50; N, 11.79.

**9c**: mp. 208–209°C; IR (KBr) ν_max_/cm^-1^ 1608 (C = N); ); ^1^H NMR (DMSO-*d*_*6*_) δ 2.26-2.28 (m, 2H), 4.19-4.22 (m, 4H), 6.86-6.89 (t, 2H, *J* = 9 Hz), 7.04-7.06(d, 2H, *J* = 9 Hz), 7.21-7.24 (t, 2H, *J* = 9 Hz), 7.54-7.56 (d, 2H, *J* = 9 Hz), 7.89-7.91 (d, 4H), 8.30 (s, 2H), 8.50-8.51 (d, 2H, *J* = 9 Hz). MS *m/z* (*%*) 462 (M^+^). For C_27_H_22_N_6_O_2_ Calcd: C, 70.12; H, 4.79; N, 18.17. Found: C, 70.14; H, 4.77; N, 18.16.

**9d**: mp. 273–275°C; IR (KBr) ν_max_/cm^-1^ 1608 (C = N); ); ^1^H NMR (DMSO-*d*_*6*_) δ 1.93-1.95 (m, 4H), 4.15-4.18 (m, 4H), 7.14-7.17(d, 4H, *J* = 9 Hz), 7.38-7.43(t, 2H, *J* = 9 Hz), 7.83-7.91 (m, 6H, *J* = 9 Hz), 8.67(s, 2H), 8.82-8.84 (d, 2H, *J* = 9 Hz); ^13^C NMR (DMSO-*d*_*6*_) δ 25.26, 67.41, 109.65, 112.14, 114.50, 115.24, 116.31, 120.57, 128.46, 136.91, 140.62, 159.86. MS *m/z* (*%*) 476 (M^+^). For C_28_H_24_N_6_O_2_ Calcd: C, 70.57; H, 5.08; N, 17.64. Found: C, 70.54; H, 5.10; N, 17.66.

**10:** mp. ˃300°C; IR (KBr) ν_max_/cm^-1^ 1609 (C = N); ); ^1^H NMR (DMSO-*d*_*6*_) δ 5.25 (s, 6H), 7.14-7.17(d, 6H, *J* = 9 Hz), 7.14-7.17(m, 6H), 7.96 (m, 9H), 7.83-7.91 (d, 3H, *J* = 9 Hz), 8.63(s, 3H), 9.02 (d, 3H, *J* = 9 Hz). MS *m/z* (*%*) 744 (M^+^). For C_48_H_36_N_6_O3 Calcd: C, 77.40; H, 4.87; N, 11.28. Found: C, 77.38; H, 4.85; N, 11.25.

#### Synthesis of carbonyl analogue bis(imidazopyridine) derivatives 14a,b and 15a,b

##### General procedure

A mixture of 2-aminopyridine (10 mmol), in dry benzene, dimethylformamide dimethylacetal (DMF-DMA) (30 mmol) was heated at refluxing temperature for 8h. The solvent was then removed in *vacuo* and the remaining oil was dried, then added to the appropriate bis(*ω*-bromoacetophenone) derivative (5 mmol), in absolute EtOH. The mixture was heated at refluxing temperature for 7h. The reaction mixture was then allowed to cool and the resulting solid was collected by filtration, washed thoroughly with ethanol and dried. Recrystallization from EtOH/DMF to afford the corresponding carbonyl analogue of the fused heterocyclic derivatives **14a,b** and **15a,b**.

**14a**: mp. 295–297°C; IR (KBr) ν_max_/cm^-1^ 1710 (C = O); ^1^H NMR (DMSO-*d*_*6*_) δ 4.81 (s, 4H), 7.14-7.17 (t, 2H, *J* = 9 Hz), 7.34-7.43 (t, 2H, *J* = 9 Hz), 7.43-7.45 (d, 2H, *J* = 9 Hz), 7.50-7.52 (t, 2H, *J* = 9 Hz), 7.72-7.74 (d, 2H, *J* = 9 Hz), 7.79-7.81 (t, 2H, *J* = 9 Hz), 7.86-7.87 (d, 4H, *J* = 9 Hz), 8.53 (s, 2H). MS *m/z* 502 (M^+^). For C_30_H_22_N_4_O_4_ Calcd: C, 71.70; H, 4.41; N, 11.15. Found: C, 71.72; H, 4.39; N, 11.13.

**14b**: mp. 263–265°C; IR (KBr) ν_max_/cm^-1^ 1682 (C = O); ); ^1^H NMR (DMSO-*d*_*6*_) δ 2.61-2.62 (m, 4H), 4.37-4.38 (m, 4H), 7.01-7.11(t, 2H, *J* = 9 Hz), 7.20-7.22(d, 2H, *J* = 9 Hz), 7.36-7.39 (t, 2H, *J* = 9 Hz), 7.46-7.48(m, 4H), 7.89-7.96 (m, 4H), 8.64(s, 2H), 8.98-8.99 (d, 2H, *J* = 9 Hz); ^13^C NMR (DMSO-*d*_*6*_) δ 25.31, 68.20, 111.80, 112.04, 112.72, 113.35, 114.71, 116.73, 120.62, 127.67, 128.84, 131.29, 131.64, 133.02, 139.22, 144.06, 155.70, 185.70. MS *m/z* 530 (M^+^). For C_32_H_26_N_4_O_4_ Calcd: C, 72.44; H, 4.94; N, 10.56. Found: C, 72.42; H, 4.91; N, 10.55.

**15a**: mp. 213-214°C; IR (KBr) ν_max_/cm^-1^ 1660 (C = O); ^1^H NMR (DMSO-*d*_*6*_) δ 2.27 (m, 2H), 4.26 (m, 4H), 7.16-7.19 (d, 4H, *J* = 9 Hz), 7.47 (t, 2H, *J* = 9 Hz), 7.89-7.95 (t, 2H, *J* = 9 Hz), 8.76 (s, 2H), 8.89 (d, 2H, *J* = 9 Hz). MS *m/z* 516 (M^+^). For C_31_H_24_N_4_O_4_ Calcd: C, 72.08; H, 4.68; N, 10.85. Found: C, 72.09; H, 4.65; N, 10.83.

**15b** mp. 249–250°C; IR (KBr) ν_max_/cm^-1^ 1658 (C = O); ^1^H NMR (DMSO-*d*_*6*_) δ 1.95 (m, 2H), 4.18 (m, 4H), 7.12 (d, 4H, *J* = 9 Hz), 7.26 (t, 2H, *J* = 9 Hz), 7.66 (t, 2H, *J* = 9 Hz), 7.87 (t, 4H, *J* = 9 Hz), 8.25 (s, 2H), 8.57-8.60 (d, 2H, *J* = 9 Hz). MS *m/z* 530 (M^+^). For C_32_H_26_N_4_O_4_ Calcd: C, 72.44; H, 4.94; N, 10.56. Found: C, 72.42; H, 4.92; N, 10.52.

## Competing interests

The author declare that he has no competing interests.

## Supplementary Material

Additional file 1 ^**1**^**H NMR spectra of the synthesized compounds.** Click here for file

## References

[B1] Katritzky AR Xu YJ Tu H Regiospecific synthesis of 3-substituted imidazo[1,2-*a*]pyridines, imidazo[1,2-*a*]pyrimidines, and imidazo[1,2-*c*]pyrimidines J Org Chem 2003 68 4935 4937 and references cited therein 10.1021/jo026797p 12790603

[B2] Rival Y Grassy G Michel G Synthesis and antibacterial activity of some imidazo[1,2-*a*]pyrimidine Chem Pharm Bull 1992 40 1170 1176 10.1248/cpb.40.1170 1394630

[B3] Fisher MH Lusi A Imidazo[1,2-*a*]pyridine anthelmintic and antifungal agents J Med Chem 1972 15 982 985 10.1021/jm00279a026 5065787

[B4] Hamdouchi C de Blas J del Prado M Gruber J Heinz BA Vance L 2-Amino-3-substituted-6-[(*E*)-1-phenyl-2-(*N*-methylcarbamoyl)vinyl]imidazo-[1,2-a]pyridines as a novel class of inhibitors of human rhinovirus: Stereospecific synthesis and antiviral activit*y* J Med Chem 1999 42 50 59 10.1021/jm9810405 9888832

[B5] Lhassani M Chavignon O Chezal JM Teulade JC Chapat JP Snoeck R Andrei G Balzarini J Clercq E De Gueiffier A Synthesis and antiviral activity of imidazo[1,2-*a*]pyridines Eur J Med Chem 1999 34 271 274 10.1016/S0223-5234(99)80061-0

[B6] Gueiffier A Lhassani M Elhakmaoui A Snoeck R Andrei G Chavignon O Teulade JC Kerbal A Essassi EM Debouzy JC Witvrouw M Blache Y Balzarini J De Clercq E Chapat JP Synthesis of acyclo-*C*-nucleosides in the imidazo[1,2-*a*]pyridine and pyrimidine series as antiviral agents J Med Chem 1996 39 2856 2859 10.1021/jm9507901 8709116

[B7] Rupert KC Henry JR Dodd JH Wadsworth SA Cavender DE Olini GC Fahmy B Siekierka J Imidazopyrimidines, potent inhibitors of p38 MAP kinase Bioorg Med Chem Lett 2003 13 347 350 10.1016/S0960-894X(02)01020-X 12565927

[B8] Spitzer WA Victor F Pollock GD Hayes JS Imidazo[1,2-*a*]pyrimidines and imidazo[1,2-*a*]pyrazines: the role of nitrogen position in inotropic activity J Med Chem 1988 31 1590 1595 10.1021/jm00403a018 3397997

[B9] Tully WR Gardner CR Gillespie RJ Westwood R 2-(Oxadiazolyl)- and 2-(thiazolyl)imidazo[1,2-*a*]pyrimidines as agonists and inverse agonists at benzodiazepine receptors J Med Chem 1991 34 2060 2067 10.1021/jm00111a021 1648620

[B10] Sanfilippo PJ Urbanski M Press JB Dubinsky JB Moore JB Synthesis of (aryloxy)alkylamines.2. Novel imidazo-fused heterocycles with calcium channel blocking and local anesthetic activity J Med Chem 1988 31 2221 2227 10.1021/jm00119a026 3184128

[B11] Diana P Carbone A Barraja P Kelter G Fiebig H Cirrincione G Synthesis and antitumor activity of 2,5-bis(3′-indolyl)-furans and 3,5-bis(3′-indolyl)-isoxazoles, nortopsentin analogues Bioorg Med Chem 2010 18 4524 4529 10.1016/j.bmc.2010.04.061 20472437

[B12] Toyota K Okada K Katsuta H Morita N Preparations of bis[2-(2-arylethynyl)-3-thienyl]arenes and bis[2-{2-(trimethylsilyl)ethynyl}-3-thienyl]arenes Tetrahedron 2009 65 145 151 10.1016/j.tet.2008.10.088

[B13] Todd EM Immerman ZSC Bis-ureidodeazapterin (Bis-DeAP) as a general route to supramolecular star polymers Tetrahedron 2008 64 8558 8570 10.1016/j.tet.2008.05.076

[B14] Diana P Carbone A Barraja P Montalbano A Martorana A Dattolo G Gia O Dalla Via L Cirrincione G Synthesis and antitumor properties of 2,5-bis(3′-indolyl)thiophenes: Analogues of marine alkaloid nortopsentin Bioorg Med Chem Lett 2007 17 2342 2346 10.1016/j.bmcl.2007.01.065 17306531

[B15] Blanco G Quintela JM Peinador C Efficient one-pot preparation of bis(pyrazino[2′,3′:4,5]thieno[3,2-*d*]pyrimidin-4-yl)benzenes based on an aza–Wittig/mediated annulation strategy Tetrahedron 2007 63 2034 2041 10.1016/j.tet.2006.12.049

[B16] Promarak V Punkvuang A Jungsuttiwong S Saengsuwan S Sudyoadsuk T Keawin T Synthesis, optical, electrochemical, and thermal properties of α, α′-bis(9,9-bis-n-hexylfluorenyl)-substituted oligothiophenes Tetrahedron Lett 2007 48 3661 3665 10.1016/j.tetlet.2007.03.131

[B17] Gregson SJ Howard PW Thurston DE Synthesis of the first examples of A-C8/C-C2 amide-linked pyrrolo[2,1-c][1,4]benzodiazepine dimers Bioorg Med Chem Lett 2003 13 2277 2280 10.1016/S0960-894X(03)00436-0 12824017

[B18] Shaker RM One-pot synthesis of novel 1,1′- and 1,4-bridged bis-thiazolidinone derivatives and their antimicrobial activity Phosphorus, Sulfur, Silicon and the Relat. Elem 1999 149 7 14 10.1080/10426509908037017

[B19] Diana P Carbone A Barraja P Martorana A Gia O Dalla Via L Cirrincione G 3,5-Bis(3’-indolyl)pyrazoles, analogues of marine alkaloid nortopsentin: Synthesis and antitumor properties. Bioorg Med Chem Lett 2007 17 6134 6137 10.1016/j.bmcl.2007.09.042 17911018

[B20] Carbone A Parrino B Barraja P Spano V Cirrincione G Diana P Maier A Kelter G Fiebig HH Synthesis and antiproliferative activity of 2,5-bis(3′-indolyl)pyrroles, analogues of the marine alkaloid nortopsentin Mar Drugs 2013 11 643 654 10.3390/md11030643 23455514 PMC3705363

[B21] Carbone A Spanò V Parrino B Ciancimino C Attanasi OA Favi G A facile synthesis of deaza-analogues of the bisindole marine alkaloid topsentin Molecules 2013 18 2518 2527 10.3390/molecules18032518 23442928 PMC6269752

[B22] Diana P Carbone A Barraja P Montalbano A Parrino B Lopergolo A Pennati M Zaffaroni N Cirrincione G Synthesis and antitumor activity of 3-(2-phenyl-1,3-thiazol-4-yl)-1*H*-indoles and 3-(2-phenyl-1,3-thiazol-4-yl)-1*H*-7-azaindoles ChemMedChem 2011 6 1300 1309 10.1002/cmdc.201100078 21523912

[B23] Gueiffier A Mavel S Lhassani M Elhakmaoui A Snoeck R Andrei G Chavignon O Teulade J-C Witvrouw M Balzarini J De Clercq E Chapat J-P Synthesis of imidazo[1,2-*a*]pyridines as antiviral agents J Med Chem 1998 41 5108 5112 10.1021/jm981051y 9836626

[B24] Enguehead C Renou J-N Allouchi H Leger J-M Gueiffier A Synthesis of diaryl-substituted imidazo[1,2-*a*]pyridines designed as potential Aromatase inhibitors Chem Pharm Bull 2000 48 935 940 10.1248/cpb.48.935 10923819

[B25] Donora N Laquintana V Pisu MG Dore R Murru L Latrofa A Trapani G Sanna E 2-Phenyl-imidazo[1,2-*a*]pyridine compounds containing hydrophilic groups as potent and selective ligands for peripheral benzodiazepine receptors: synthesis, binding affinity and electrophysiological studies J Med Chem 2008 51 6876 6888 10.1021/jm8006728 18834105

[B26] Lacerda RB de Lima CKF da Silva LL Romeiro NC Miranda ANP Barreiro EJ Fraga CAM Discovery of novel analgesic and anti-inflammatory 3-arylamine-imidazo[1,2-*a*]pyridine symbiotic prototypes Bioorg Med Chem 2009 17 74 84 10.1016/j.bmc.2008.11.018 19059783

[B27] Kamal A Reddy JS Ramaiah MJ Dastagiri D Bharathi EV Sagar MVP Pushpavalli SN CVL Ray P Pal Bhadra M Design, synthesis and biological evaluation of imidazopyridine/pyrimidine-chalcone derivatives as potential anticancer agents Med Chem Comm 2010 1 355 360 10.1039/c0md00116c

[B28] Ide S Katou K Itou T Motokawa C Chiyomaru Y Matsuda Y Synthesis of imidazo[1,2-*a*]pyridine derivatives and their reactions Yakugaku Zasshi 1993 113 861 869 8301539 10.1248/yakushi1947.113.12_861

[B29] Wang J Mason R VanDerveer D Feng K Bu XR Convenient preparation of a novel class of imidazo[1,5-a]pyridines: decisive role by ammonium acetate in chemoselectivity J Org Chem 2003 68 5415 5418 10.1021/jo0342020 12816512

[B30] Adib M Sheibani E Zhu L-G Mirzaei P An efficient synthesis of 3-amino-2-arylimidazo[1,2-*a*]pyridines Tetrahedron Lett 2008 49 5108 5110 10.1016/j.tetlet.2008.05.134

[B31] Clements Jewery S Danswan G Gardner CR Matharu SS Murdoch R Tully WR Westwood R (Imidazo[1,2-*a*]pyrimidin-2-yl)phenylmethanones and related compounds as potential nonsedative anxiolytics J Med Chem 1988 31 1220 1226 10.1021/jm00401a025 2897468

[B32] Jiang B Shi F Tu SJ Microwave-assisted multicomponent reactions in the heterocyclic chemistry Curr Org Chem 2010 14 357 378 10.2174/138527210790231892

[B33] Kappe CO Microwave dielectric heating in synthetic organic chemistry Chem Soc Rev 2008 37 1127 1139 10.1039/b803001b 18497926

[B34] Dallinger D Kappe CO Microwave-assisted synthesis in water as solvent Chem Rev 2007 107 2563 2591 10.1021/cr0509410 17451275

[B35] Elwahy AHM Darweesh AF Shaaban MR Microwave-assisted synthesis of bis(enaminoketones): versatile precursors for novel bis(pyrazoles) via regioselective 1,3-dipolar cycloaddition with nitrileimines J Heterocycl Chem 2012 49 1120 1125 10.1002/jhet.952

[B36] Shaaban MR El-Sayed R Elwahy AHM Construction of fused heterocycles by metal-mediated [2 + 2 + 2]cyclotrimerization of alkynes and/or nitriles Tetrahedron 2011 67 6095 6130 10.1016/j.tet.2011.04.096

[B37] Shaaban MR Saleh TS Mayhoub AS Farag AM Single step synthesis of new fused pyrimidine derivatives and their evaluation as potent Aurora-A kinase inhibitors Eur J Med Chem 2011 46 3690 3695 10.1016/j.ejmech.2011.05.033 21664013

[B38] Elwahy AHM Shaaban MR Synthesis of trifluoromethyl-substituted fused bicyclic heterocycles and their corresponding benzo-fused analogues Curr Org Synthesis 2010 7 433 454 10.2174/157017910792246117

[B39] Shaaban MR Elwahy AHM Bis(a-bromo ketones): versatile precursors for novel bis(s-triazolo[3,4-*b*][1,3,4]thiadiazines) and bis(as-triazino[3,4-*b*][1,3,4]thiadiazines) J Heterocycl Chem 2012 49 640 645 10.1002/jhet.861

[B40] Elwahy AHM Difunctional heterocycles: A convenient synthesis of bis(4,5-dihydropyrazolyl)ethers from their precursors bis(chalcones) J Chem Res (S) 1999 602 603

[B41] Tani H Murayama K Toda F Oligomers and polymers containing triple bonds. III. derivatives of α, *ω*-bis(4-ethynylphenoxy)alkane Bull Chem Soc Jpn 1964 37 919 923 10.1246/bcsj.37.919

