# Supplementary material for: Microwave assisted synthesis of bis and tris(ω-bromoacetophenones): versatile precursors for novel bis(imidazo[1,2-a]pyridines), bis(imidazo[1,2-a]pyrimidines) and their tris-analogs
Source: Chem Cent J. 2013 Jun 19;7:105. doi: 10.1186/1752-153X-7-105 (PMC3698004; doi:10.1186/1752-153X-7-105)
Supplement: Additional file 1 — 1H NMR spectra of the synthesized compounds. [file 1752-153X-7-105-S1.docx]

**Additional file 1**

**^
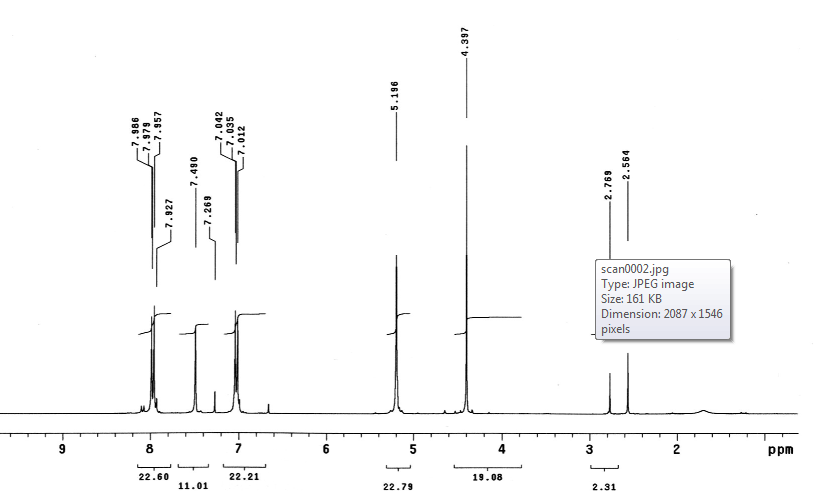
^**

**^1^H NMR spectra of compound 6**

**^
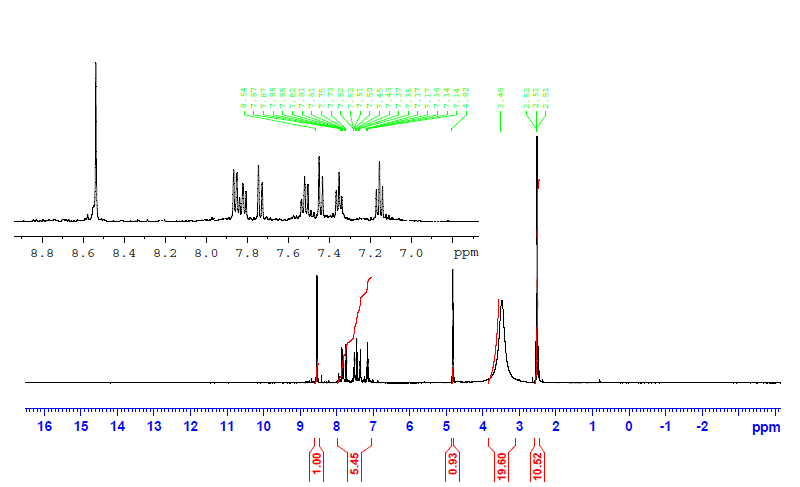
^**

**^1^H NMR spectra of compound 8a**

**^
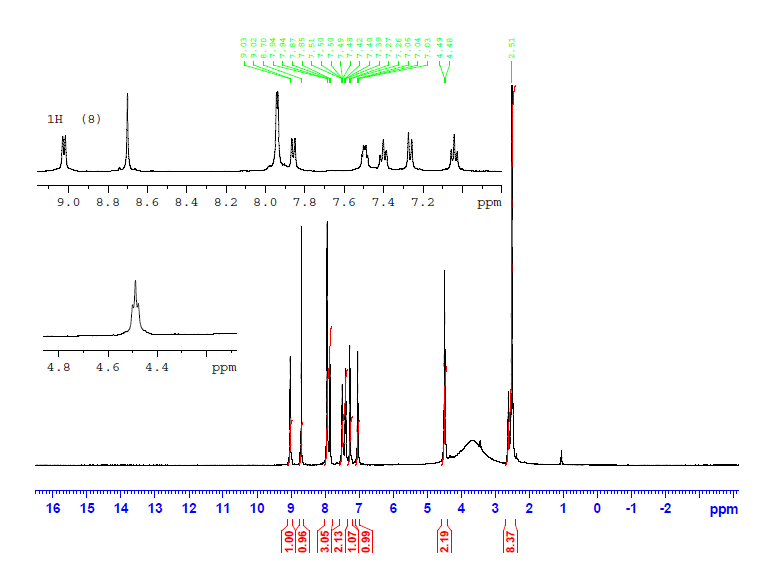
^**

**^1^H NMR spectra of compound 8b**

**^
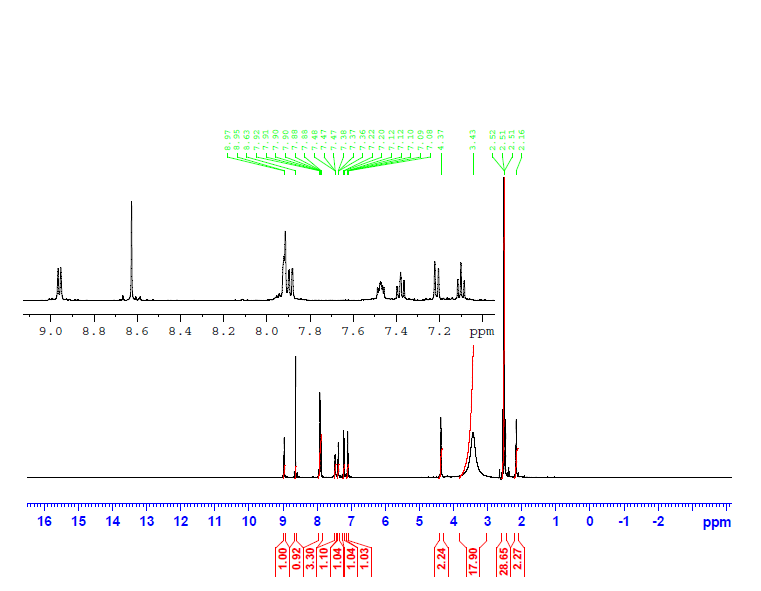
^**

**^1^H NMR spectra of compound 8c**

**^
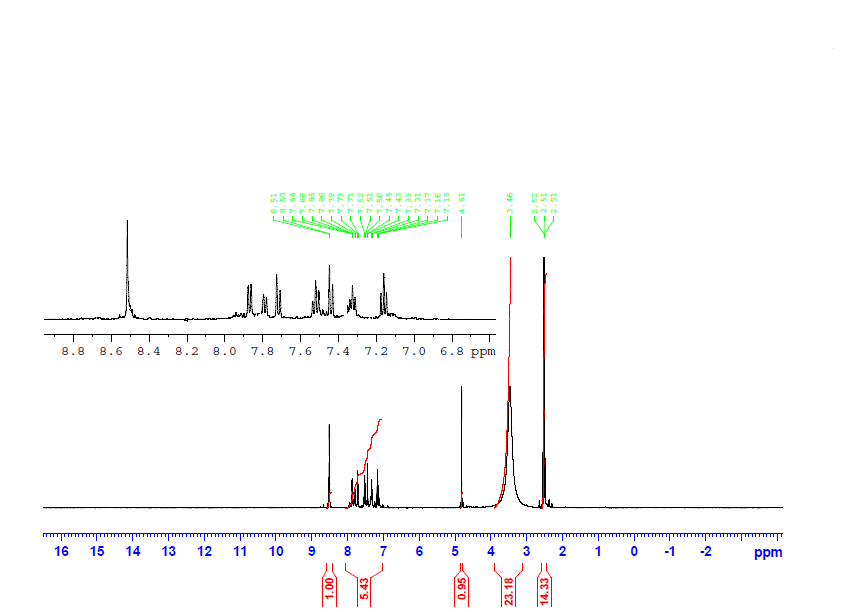
^**

**^1^H NMR spectra of compound 8d**

**^
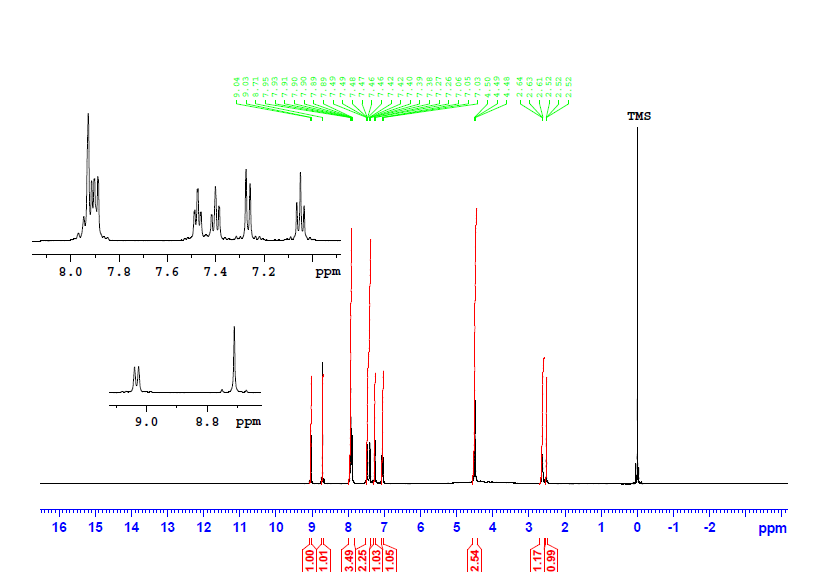
^**

**^1^H NMR spectra of compound 8e**

**^
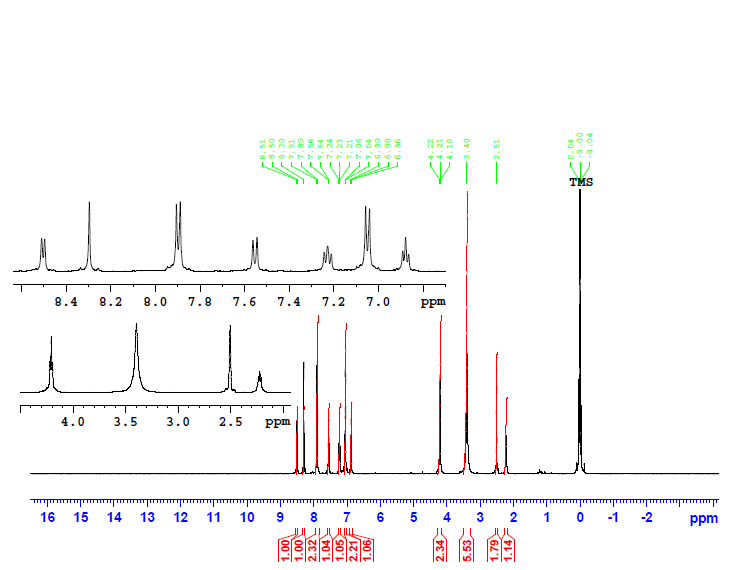
^**

**^1^H NMR spectra of compound 9a**

**^
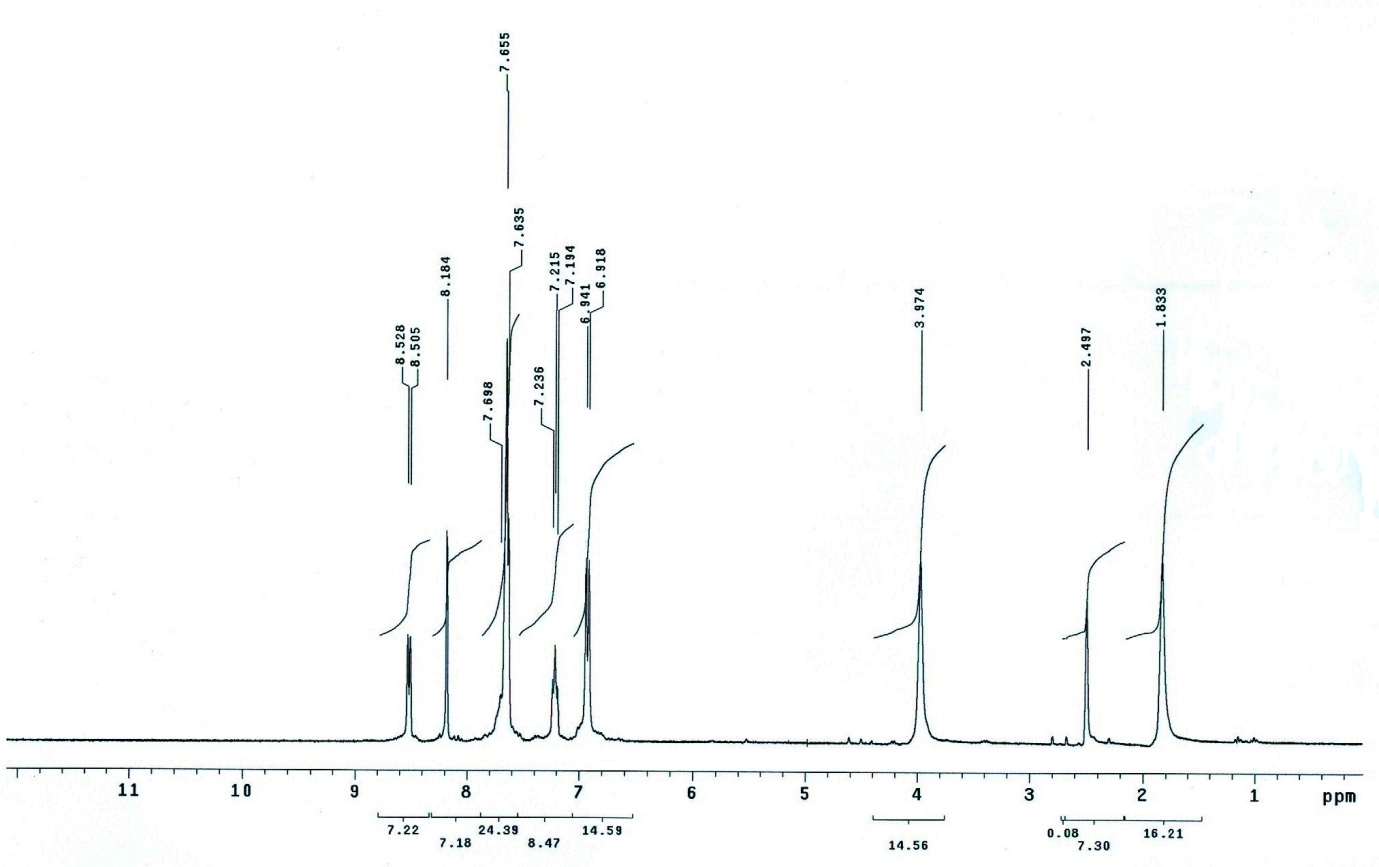
^**

**^1^H NMR spectra of compound 9b**

**^
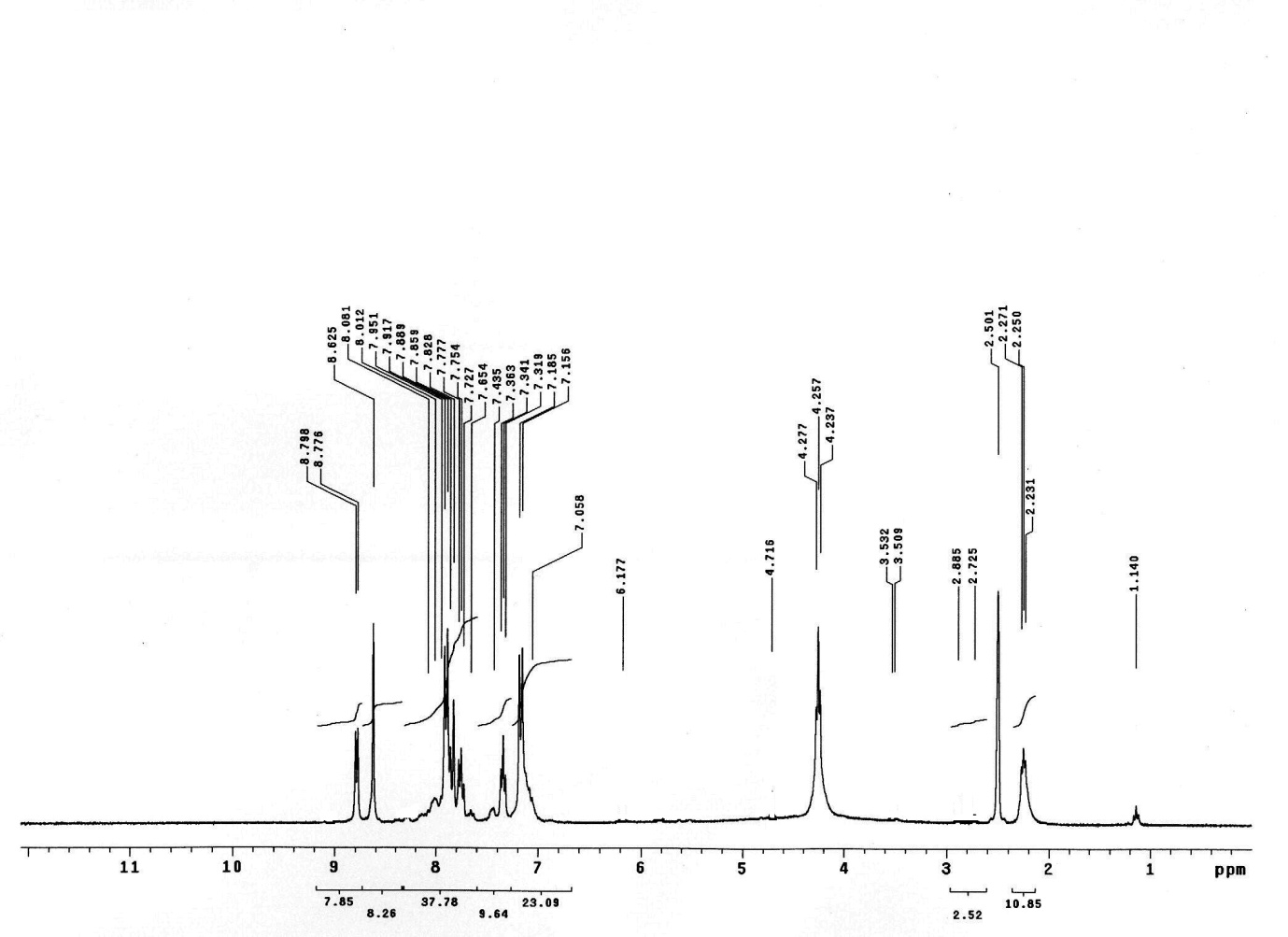
^**

**^1^H NMR spectra of compound 9c**

**^
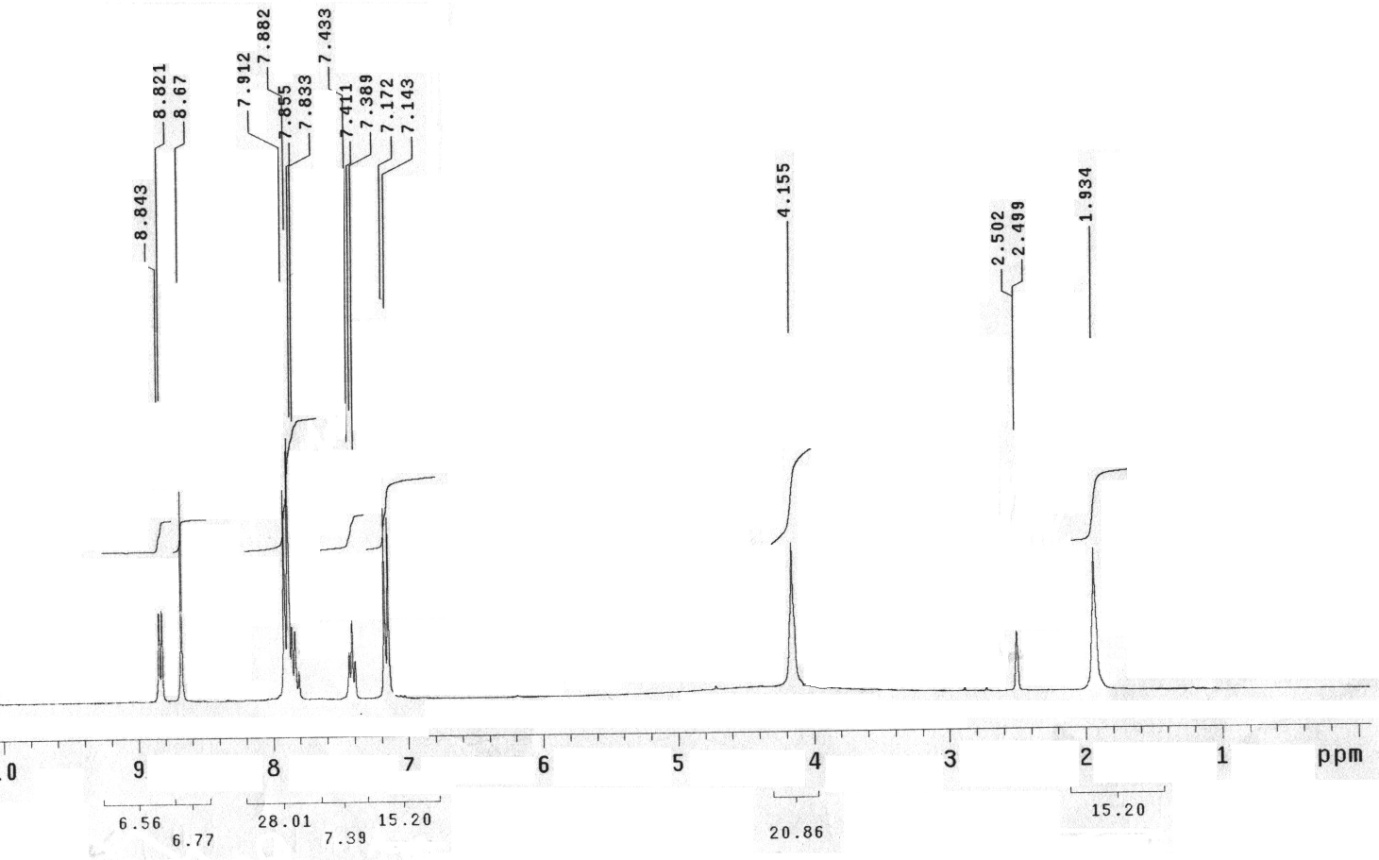
^**

**^1^H NMR spectra of compound 9d**

**^
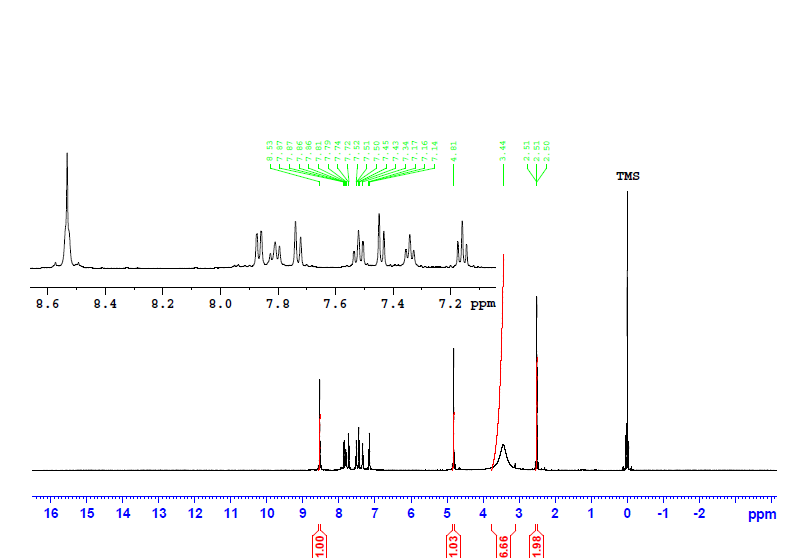
^**

**^1^H NMR spectra of compound 14a**

**^
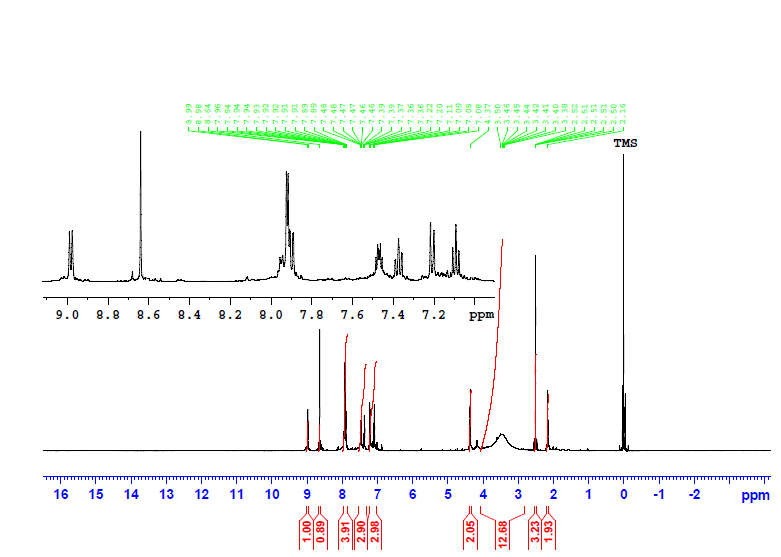
^**

**^1^H NMR spectra of compound 14b**

**^
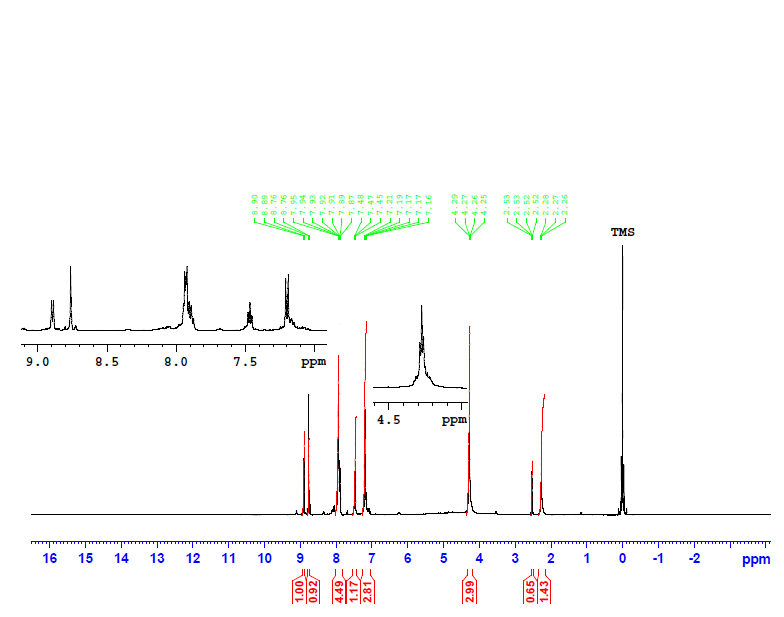
^**

**^1^H NMR spectra of compound 15a**

**^
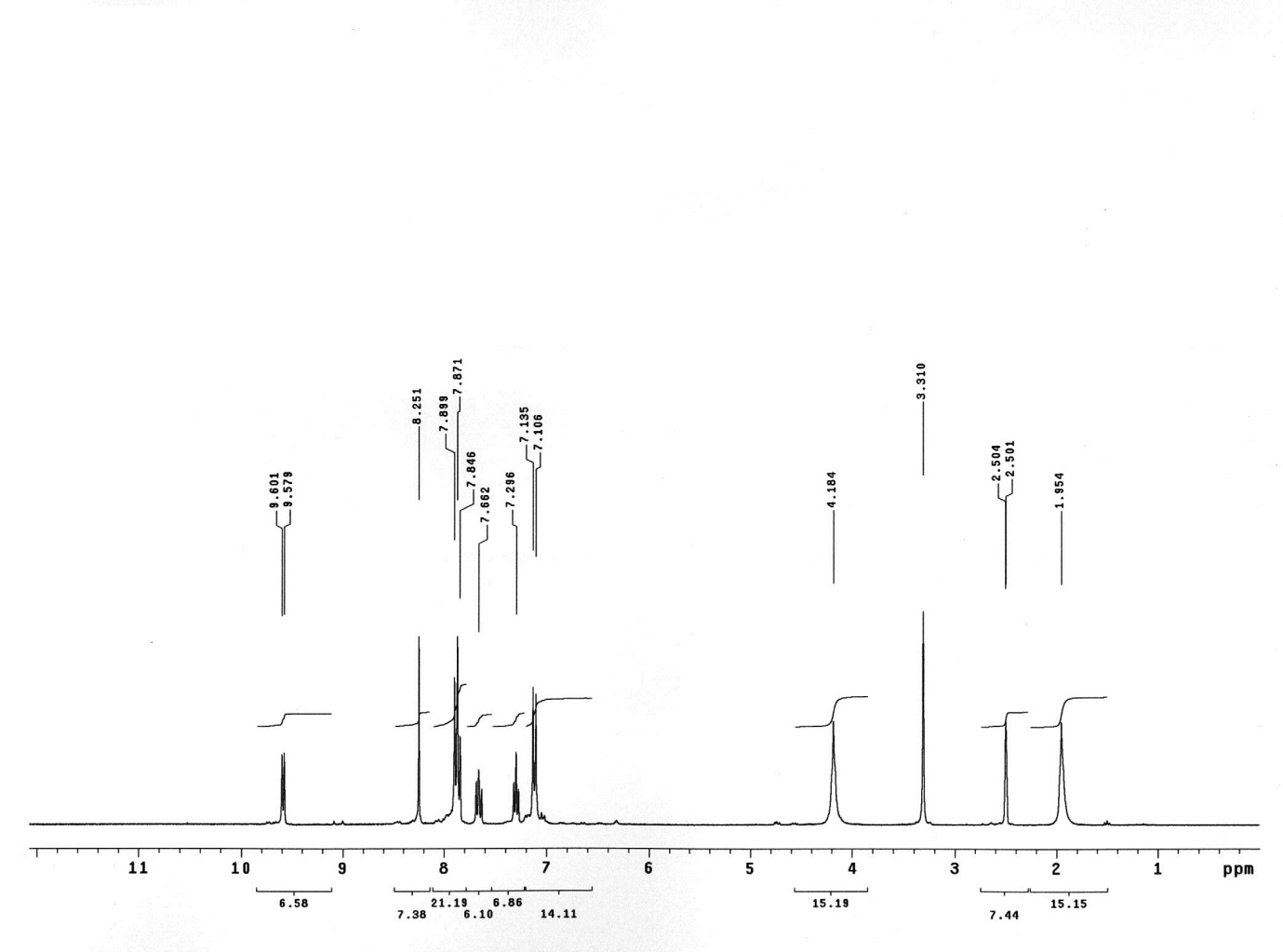
^**

**^1^H NMR spectra of compound 15b**
